# Outcome after Elective Percutaneous Coronary Intervention Depends on Age in Patients with Stable Coronary Artery Disease – An Analysis of Relative Survival in a Multicenter Cohort and an OCT Substudy

**DOI:** 10.1371/journal.pone.0154025

**Published:** 2016-04-22

**Authors:** Christian Roth, Clemens Gangl, Daniel Dalos, Lisa Krenn, Sabine Scherzer, Anna Gerken, Martin Reinwein, Chao Zhang, Michael Hagmann, Thomas Wrba, Georg Delle-Karth, Thomas Neunteufl, Gerald Maurer, Paul Vock, Harald Mayr, Bernhard Frey, Rudolf Berger

**Affiliations:** 1 Department of Internal Medicine II, Cardiology, Medical University of Vienna, Vienna, Austria; 2 Section of Medical Statistics, Medical University of Vienna, Vienna, Austria; 3 Section of Medical Information and Retrieval Systems, Department of Informatics and Intelligent Systems, Medical University of Vienna, Vienna, Austria; 4 Department of Internal Medicine IV, Cardiology, Hospital of Hietzing, Vienna, Austria; 5 Department of Internal Medicine I, Cardiology, University Hospital of Krems, Krems an der Donau, Austria; 6 Karl Landsteiner Private University for Health Sciences, Krems an der Donau, Austria; 7 Department of Internal Medicine III, Cardiology, University Hospital of St. Pölten, St. Pölten, Austria; 8 Department of Internal Medicine I, Cardiology and Nephrology, Hospital of St. John of God, Eisenstadt, Austria; Scuola Superiore Sant'Anna, ITALY

## Abstract

**Background:**

Age is a strong predictor of survival in patients with coronary artery disease. In elder patients with increasing co-morbidities percutaneous coronary intervention (PCI) is associated with more complications and worse outcome. The calculation of relative survival rates adjusts for the “background” mortality in the general population by correcting for age and gender. We analyzed if elder patients after elective PCI have a worse relative survival compared to younger patient groups.

**Methods:**

A total of 8,342 patients who underwent elective PCI at two high volume centers between 1998 and 2009 were analyzed.

**Results:**

The survival of our patients after PCI (observed survival) was slightly lower compared to the general population (expected survival) resulting in a slightly decreasing relative survival curve. In a multivariate Cox regression model age amongst others was a strong predictor of survival. Stratifying patients according to their age the relative survival curves of younger patients (Quartile 1: <58 years; 2,046 patients), elder patients (Quartile 3: 66–73 years; 2,090 patients) and very old patients (Quartile 4: >73 years; 2,307 patients) were similar. The relative survival of mid-aged patients (Quartile 2: 58–65 years; 1,899 patients) was better than that of all other patient groups. The profile of cardiovascular risk factors differs between the various groups resulting in different composition and burden of coronary plaques in an optical coherence tomography sub-study.

**Conclusion:**

Patients after elective PCI have a slightly worse long-term survival compared to the age- and sex-matched general population. This is also true for different groups of age except for mid-aged patients between 58 and 63 years. Elder patients between 66 and 73 years and above 73 years have a similar relative survival compared to younger patients below 58 years, and might therefore have similar benefit from elective PCI.

## Introduction

Elder patients with stable coronary artery disease (CAD) often suffer from multiple co-morbidities and are consequently more fragile. Accordingly, these patients experience increased rates of in-hospital complications like death, Q wave myocardial infarction (MI), stroke, renal failure and vascular complications after Percutaneous Coronary Intervention (PCI) [1,2]. In elder patients interventional complications were demonstrated to be the strongest predictor of hospital mortality after PCI [3]. Therefor, elder patients with CAD face significantly higher initial [4,5] but also long-term mortality [4,6] after catheterization. Recent guidelines point out this higher risk of complications during and after coronary revascularization in elder patients [7].

The feasibility to perform PCI safely has improved due to new techniques and treatment strategies. Accordingly, recent studies have demonstrated substantially higher technical success rates and considerably lower acute complication rates for coronary angioplasty compared with earlier years [8–13]. This is true especially in elder patients: improvements have been achieved despite the increase of the average age of elder patients admitted for PCI and despite the growing extent of CAD in these patients [8]. As a result, an increasing number of elder patients with more severe CAD nowadays undergo treatment by PCI [3,4,14–17]. However, recent guidelines still indicate that this group of patients is undertreated and under-represented in clinical trials [7].

In general, age per se is a strong predictor of mortality as elder people are at higher risk to die than younger people. Similarly in patients with CAD, higher age besides other factors is a strong predictor of outcome [2]. Accordingly, specific statistical assumptions are prerequisite for correct comparison of outcome between younger and elder patients after elective PCI. The calculation of relative survival is a statistical method, which adjusts the mortality rate of a patient population with a certain disease for the “background” mortality in the general population, to evaluate the mortality rate caused by this certain disease. The survival rate adjusted for this background mortality is called relative survival. Thereby, this method allows comparing patients in different groups of age in regard to differences in relative survival. The aim of this study is to compare the relative survival of patients in different groups of age with stable CAD, who underwent coronary intervention.

## Methods

### Study population

This multicenter observational cohort study retrospectively included consecutive patients who underwent elective PCI at two high volume centers (Medical University of Vienna [MUW], University Hospital of St. Pölten) between January 1^st^, 1998 and December 31^st^, 2009. Patients with an acute coronary syndrome were excluded from our analysis. Most of these patients suffered from a STEMI or NSTEMI and underwent acute coronary angiography. However, we also excluded patients with unstable angina class I to III [18]. Patients with significant and symptomatic valve disease and significant coronary artery disease did not undergo elective PCI but were assigned to valve repair or replacement and coronary artery bypass grafting. Between 2007 and 2009 a few patients with significant aortic stenosis underwent elective PCI before Transcatheter Aortic Valve Implantation. These patients were excluded from analysis.

Experienced interventionists approved all coronary angiograms. The coronary angioplasty approach and medical treatment were consistent with contemporary practices. The survival status of all patients included in the database was prospectively retrieved from the Austrian Death Registry database (Statistik Austria) at the due day (October 31^st^, 2010). After clarification of the survival status, patients were anonymized in the electronic data files by receiving a unique patient identification (ID). This patient identification was used for all analyses. The study is in line with the Declaration of Helsinki and was approved by the ethics committee of the MUW and the federal state of Lower Austria in St. Pölten. According to the ethics committee an informed consent was not required because of the retrospective inclusion of patients and analysis of their data in an anonymous format.

### Data collection

Eligible patients were identified in the corresponding database of each catheter laboratory (MUW—Cardio-Report; University Hospital St. Pölten—Cathlab). Study data including baseline characteristics, co-morbidities, angiographic characteristics, and interventional results were extracted from the hospital information system (Krankenhausinformationssystem—KIS). In addition, re-angiograms were documented for each patient. All data were gathered in a comprehensive database, which had been established in co-operation with the Center for Medical Statistics, Informatics, and Intelligent Systems (CeMSIIS) of the MUW using the Research Documentation and Analysis (RDA) IT-system. At the end of the follow-up period, the data from the clinical database were synchronized with the Austrian Death Registry database (Statistik Austria) to check the life status and to evaluate the date and cause of death of each patient who died. This database comprises information on every case of death in Austria.

### OCT subgroup analysis—data acquisition and analysis

For plaque characterization we analyzed optical coherence tomography (OCT) images from 40 patients including 10 consecutive patients in each group of age. OCT images were performed after final stent-optimization. The OCT images were obtained using a frequency domain—OCT system (St. Jude Medical Inc., St. Paul, MN, USA). This system is equipped with a turnable laser light source with a sweep range of 1.250 to 1.370nm. The optical fibre is encapsulated within a rotating torque wire built in a rapid-exchange 2.6-F catheter. The OCT imaging catheters were delivered over a 0.014 inch (0.0356cm) guide wire through a 6-F guiding catheter. Before image acquisition OCT images were calibrated adjusting the Z-offset to obtain accurate measurements. Images were acquired using a motorized pullback system at a speed of 20mm/s during flush of 4 to 6mL/s of iso-osmolar contrast (Iodixanol 320, VisipaqueTM, GE Health Care, Cork, Ireland) through the guiding catheter to replace blood flow and permit visualization of the vessel.

**Fig 1:**
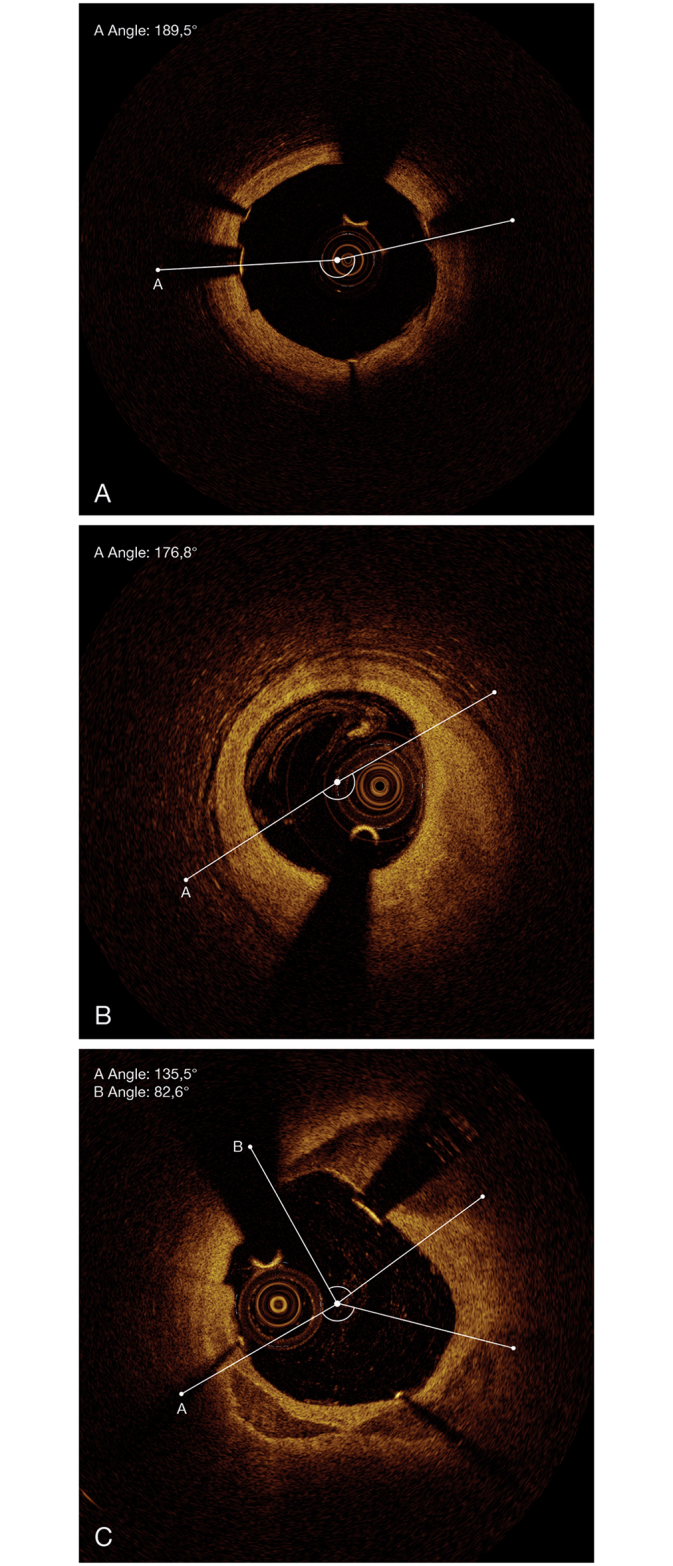
OCT—examples of plaque. **A**. fibrous plaque. **B**. lipid plaque. **C**. calcified plaque.

Plaque characteristics were measured manually by two independent observers. If there was disagreement between the observers, a consensus was made regarding the measurements of the plaque. A lipid plaque was defined as a low signal region with diffuse border and a maximum arc of lipid ≥ 90°. This maximum arc is an excellent discriminator of fibroatheroma [19] and together a fibrous cap thickness ≤85 μm identifies thin-cap fibroatheroma when combined with a fibrous cap thickness ≤85 μm [20]. Meassurements of fibrous cap thickness were not possible, as OCT recordings were performed after stenting. A calcified plaque was defined as a signal-poor or heterogeneous region with a sharply delineated border, an arc of calcification ≥ 40°, and the absence of a lipid plaque. Plaques, presenting as a homogenous, high backscattering region, were defined as fibrous plaques. In addition, also mixed types of plaque without fulfilling the criteria for a lipid or calcified plaque were classified as fibrous plaque. The arc angle (°) was measured with a protractor centered on the lumen. For calculation of lipid, calcification, and total plaque index we measured the arc of the according plaque in every cross-sectional frame (every 200 μm) and multiplied the averaged arc by the length of the plaque. Maximal plaque thickness (mm) was measured as the maximum distance occupied by the plaque in a line extrapolated from the centre of the vessel lumen. Maximal cross-sectional area (mm^2^) was measured at the same place by tracing the outline of the plaque. In lipid plaques with considerable amount of fibrous content maximal cross-sectional area and maximal plaque thickness were evaluated in the fibrous section of the plaque. Examples for lipid, fibrous and calcified plaques are shown in Fig 1.

### Primary outcome

In our analysis the primary outcome was defined as survival time after elective PCI—both observed and relative (as compared with a matched normal population). Whereas observed survival gives the rate of surviving patients in an observed group, relative survival compares observed survival with the survival expected according to the age-, sex- and follow-up year-matched population estimated from the life table provided by Statistik Austria.

### Statistical methods

Continuous variables are presented as mean ± SD or median with interquartile range (IQR) as appropriate. Categorical variables are presented as counts or percentages as appropriate. Continuous variables were compared using a 1-factor analysis of variance followed by a Bonferroni procedure. Ordinal data were compared using a Kruskal-Wallis test followed by Shaffer-corrected *U* tests. Categorical data were compared using a chi-square test. To evaluate the impact of possible risk factors on survival, univariate Cox regression models were calculated. Those risk factors, which were available in a large proportion of patients (at least 4,900 patients) and showed statistically significant influence in the univariate model, were further analyzed in a multivariate model. To compare the cohort survival with the according age and gender matched background population, multiplicative relative survival models were used. This method compares patients in different groups of age (quartiles) in regard to differences in relative survival. For this comparison, online life tables provided by Statistik Austria were used [21]. Relative survival curves according to Hakulinen's method were plotted. Analysis was performed using R 3.0.1. All p-values <0.05 were considered as statistically significant. No correction for multiplicity was done.

## Results

### Baseline and angiographic characteristics

A total of 8,342 patients with stable CAD, 2,944 male and 5,398 female patients with a median age of 66 years, underwent PCI in two high volume centers (MUW n = 3,906, University Hospital St. Pölten n = 4,436) during the observational period. The patient characteristics are given in Table 1. Interestingly, 24% of patients suffered from a prior MCI, 17% had a prior PCI, and 9% had a prior coronary artery bypass graft (CABG). Arterial hypertension (70% of all patients) and treated hyperlipidemia (70%) were the most common risk factors. 25% of patients had a history of diabetes, with 18% of patients suffering from non-insulin-dependent diabetes mellitus (NIDDM) and 7% suffering from insulin-dependent diabetes mellitus (IDDM). The cardiovascular medication and angiographic characteristics are listed in Tables 2 and 3. Sixty percent of the patients had a one-vessel disease, while 18% of the patients had a two- and 22% had a three-vessel disease. About one-fifth (17%) of the patients had a chronic total occlusion. Most of the patients (6,583 patients– 79%) underwent one procedure within the observation period, whereas 1,759 (21%) patients had two or more procedures (Table 3).

**Table 1:** Baseline.

	All, n = 8,342	Quartile 1, n = 2,046	Quartile 2, n = 1,899	Quartile 3, n = 2,090	Quartile 4, n = 2,307	p
Age, years (n), median (IQR)	(8342), 66 (58/74)	(2046), 52 (46/55)	(1899), 62 (60/64)	(2090), 70 (68/71)	(2307), 78 (76/81)	<0,0001 *†‡§¶#
Gender						<0.0001 *‡§¶#
- Female, n (%)	2944 (35)	545 (27)	534 (28)	737 (35)	1,128 (49)	
- Male, n (%)	5398 (65)	1501 (73/73/28)	1365 (72/72/25)	1353 (65/65/25)	1179 (51/51/22)	
Height, cm (n), Median (IQR)	(6415), 171 (165/178)	(1575), 173 (166/180)	(1803), 173 (166/178)	(1616), 170 (165/176)	(1421), 168 (160/173)	<0.0001 †‡§¶#
Weight, kg (n), Median (IQR)	(6437), 80 (70/90)	(1577), 82 (71/94)	(1807), 83 (74/92)	(1621), 80 (71/90)	(1432), 74 (66/82)	<0.0001 †‡§¶#
Body-Mass-Index, kg/m^2^ (n), Mean±SD	(5712), 27.7±5	(1565), 27.7±5	(1540), 28.3±5	(1372), 27.8±4	(1235), 26.6±4	<0.0001 *‡§¶#
Heart rate, bpm (n), Median (IQR)	(5366), 68 (60/78)	(1472), 69 (60/79)	(1440), 67 (60/78)	(1310), 68 (60/77)	(1144), 68 (60/78)	<0.246
Arterial hypertension, n (%)	4981 (70)	1027 (56)	1397 (71)	1368 (78)	1189 (75)	<0.0001 *†‡§¶#
Systolic blood pressure, mmHg (n), Median (IQR)	(5213), 135 (120/150)	(1415), 130 (120/140)	(1409), 134 (121/149)	(1264), 139 (125/150)	(1125), 140 (125/150)	<0.0001 *†‡§¶
Diastolic blood pressure, mmHg (n), Median (IQR)	(5213), 80 (70/85)	(1415), 80 (70/88)	(1409), 80 (70/88)	(1264), 80 (70/85)	(1125), 74 (66/80)	<0.0001 †‡§¶#
Diabetes mellitus						
- NIDDM, n (%)	1255 (18)	198 (11)	363 (19)	352 (20)	342 (22)	<0.0001 *†‡¶
- IDDM, n (%)	522 (7)	103 (6)	138 (7)	155 (9)	126 (8)	0,002 †‡§
Hyperlipidemia, n (%)	5026 (70)	1171 (64)	1492 (76)	1311 (74)	1052 (66)	<0.0001 *†¶#
Total cholesterol, mg/dl (n), Median (IQR)	(2489), 185 (155/217)	(595), 192 (158/222)	(666), 191 (160/221)	(627), 178 (154/212)	(601), 178 (150/213)	<0.0001 †‡§¶
HDL, mg/dl (n), Median (IQR)	(1551), 47 (39/57)	(357), 45 (38/54)	(424), 47 (39/59)	(391), 48 (39/57)	(379), 48 (40/59)	0.003 *‡
LDL, mg/dl (n), Median (IQR)	(1562), 113 (86/141)	(363), 121 (92/151)	(425), 116 (88/142)	(392), 110 (83/138)	(382), 106 (83/133)	0.002 †‡
Serum-creatine,mg/dl (n), Median (IQR)	(2948), 1.03 (0.89/1.21)	(872), 0.95 (0.85/1.09)	(738), 1.02 (0.89/1.18)	(708), 1.05 (0.92/1.22)	(630), 1.14 (0.95/1.37)	<0.0001 *†‡¶#
Creatine-clearance **, ml/min (n), Median (IQR)	(2611), 79 (59/100)	(872), 98 (74/120)	(738), 82 (65/99)	(708), 70 (55/84)	(630), 49 (35/61)	<0.0001 *†‡§¶#
Smoker*						
- current, n (%)	1208 (17)	556 (30)	382 (20)	183 (10)	87 (6)	<0,0001 *†‡§¶#
- previous, n (%)	1539 (22)	349 (19)	494 (25)	402 (23)	294 (19)	<0.0001 *†¶#
Family history, n (%)	1220 (17)	418 (23)	324 (17)	261 (15)	217 (14)	<0.0001 *†‡¶
Central vascular disease, n (%)	893 (13)	106 (6)	217 (11)	261 (15)	309 (19)	<0.0001 *†‡§¶#
Peripheral vascular disease, n (%)	710 (10)	86 (5)	175 (9)	212 (12)	237 (15)	<0.0001 *†‡§¶#
Prior myocardial infarction, n (%)	1715 (24)	329 (18)	465 (24)	454 (26)	467 (29)	<0.0001 *†‡¶#
Prior PCI, n (%)	1194 (17)	225 (12)	362 (19)	357 (20)	250 (16)	<0.0001 *†‡¶#
Prior coronary artery bypass graft, n (%)	638 (9)	60 (3)	150 (8)	206 (12)	222 (14)	<0.0001 *†‡§¶
Pacemaker, n (%)	272 (4)	21 (1)	35 (2)	71 (4)	145 (9)	<0.0001 *†‡§¶#
CRT, n (%)	16 (0.2)	0	6 (0.3)	5 (0.3)	5 (0.3)	0.134
LVEF (levogram), % (n), Median (IQR)	(428), 56 (41/68)	(100), 60 (47/69)	(134), 58 (42/69)	(102), 55 (41/66)	(92), 51 (34/65)	0.035 ‡
NT-proBNP, pg/ml (n), Median (IQR)	(810), 595 (154/2259)	(208), 195 (72/1601)	(202), 373 (109/1417)	(183), 652 (251/2259)	(217), 1313 (406/4810)	<0.0001 ‡¶
NYHA class						<0.0001 *†‡§¶#
- Class I, n (%)	4132 (58)	1228 (67)	1160 (59)	978 (56)	766 (48)	
- Class II-III, n (%)	3017 (42)	608 (33)	798 (41)	784 (45)	827 (52)	
CCS class						<0.0001 *†‡§
- Class I, n (%)	3508 (49)	1001 (55)	974 (50)	804 (46)	729 (46)	
- Class II, n (%)	2160 (30)	512 (28)	577 (30)	575 (33)	496 (31)	
- Class III, n (%)	1481 (21)	323 (18)	407 (21)	383 (22)	368 (23)	

** calculated with the Cockcroft-Gault formula.

Significant differences according to quartiles:

* = Q1 vs. Q2,

^†^ = Q1 vs. Q3,

^‡^ = Q1 vs. Q4,

^§^ = Q2 vs. Q3,

^¶^ = Q2 vs. Q4,

^#^ = Q3 vs. Q4.

Quartile 1 = <58 years;

Quartile 2 = 58–65 years;

Quartile 3 = 66–73 years;

Quartile 4 = >73 years.

**Abbreviations**:

CCS = canadian cardiovascular society;

CRT = cardiac resynchronization therapy;

HDL = high density lipoprotein;

IDDM = insulin dependent diabetes mellitus;

LDL = low density lipoprotein;

LVEF = left ventricular ejection fraction;

NIDDM = non insulin dependent diabetes mellitus;

NT-proBNP = n-terminal pro-brain natriuretic peptide;

NYHA = new york heart association;

p = p-value;

PCI = percutaneous coronary intervention.

**Table 2:** Medical therapy.

	All, n = 8,342	Quartile 1, n = 2,046	Quartile 2, n = 1,899	Quartile 3, n = 2,090	Quartile 4, n = 2,307	p
ACE inhibitor, n (%)	3223 (52)	733 (53)	889 (50)	828 (49)	773 (52)	<0.0001 *†‡
ARB, n (%)	1504 (24)	298 (21)	426 (24)	407 (25)	373 (26)	0.016 †‡
Aldosteron-antagonist, n (%)	577 (9)	101 (7)	122 (7)	139 (9)	215 (15)	<0.0001 †‡§¶#
Beta-blocker, n (%)	4166 (67)	954 (76)	1194 (64)	1086 (65)	932 (63)	<0.0001 *†‡
Statin, n (%)	4706 (71)	1080 (66)	1420 (76)	1207 (72)	999 (68)	<0.0001 *†§¶#
Aspirin, n (%)	7001 (98)	1813 (99)	1323 (98)	1712 (97)	1553 (98)	0.004 †‡§
P2Y12 inhibitor, n (%)	7010 (98)	1800 (98)	1922 (98)	1729 (98)	1559 (98)	0.926
Dual antiplatelet therapy, n (%)	6866 (96)	1800 (98)	1868 (95)	1679 (95)	1519 (95)	<0.0001 *†‡
Calcium-channal blocker, n (%)	1637 (26)	291 (19)	489 (27)	438 (27)	419 (29)	<0.0001 *†‡
Nitrates, n (%)	1326 (22)	191 (13)	351 (20)	381 (25)	403 (29)	<0.0001 *†‡§¶#

Significant differences according to quartiles:

* = Q1 vs. Q2,

^†^ = Q1 vs. Q3,

^‡^ = Q1 vs. Q4,

^§^ = Q2 vs. Q3,

^¶^ = Q2 vs. Q4,

^#^ = Q3 vs. Q4.

Quartile 1 = <58 years;

Quartile 2 = 58–65 years;

Quartile 3 = 66–73 years;

Quartile 4 = >73 years.

**Abbreviations**:

ACE = angiotensin-converting-enzyme;

ARB = angiotensin receptor blocker;

p = p-value.

**Table 3:** Angiographic characteristics.

	All, n = 8,342	Quartile 1, n = 2,046	Quartile 2, n = 1,899	Quartile 3, n = 2,090	Quartile 4, n = 2,307	p
Vessel disease						0.482
- 1VD, n (%)	4292 (60)	1130 (62)	1149 (59)	1066 (61)	947 (59)	
- 2VD, n (%)	1270 (18)	313 (17)	375 (19)	302 (17)	280 (18)	
- 3VD, n (%)	1587 (22)	393 (21)	434 (22)	394 (22)	366 (23)	
Chronic total occlusion, n (%)	1203 (17)	287 (16)	338 (17)	308 (18)	270 (17)	0.441
Number of procedures						0.032 ‡¶#
- one, n (%)	6,583 (79)	1,599 (78)	1,486 (78)	1,620 (78)	1,878 (81)	
- two, n (%)	1,490 (18)	372 (18)	349 (18)	399 (19)	370 (16)	
- > two, n (%)	269 (3)	75 (4)	64 (4)	71 (3)	59 (3)	
Patients treated with						
- drug-eluting stent, n (%)	1699 (80)	347 (84)	470 (79)	465 (80)	417 (78)	0,142
- bare-metal stent, n (%)	427 (20)	67 (16)	126 (21)	117 (20)	117 (22)	0,142
Troponin T, ng/ml						
- before PCI (n), Median (IQR)	(144), 0.03 (0.01/0.05)	(32), 0.02 (0/0.06)	(31), 0.02 (0/0.05)	(32), 0.03 (0.02/0.04)	(49), 0.04 (0.01/0.05)	0.771
- day 1 after PCI (n), Median (IQR)	(144), 0.03 (0.01/ 0.06)	(32), 0.04 (0.01/0.07)	(31), 0.03 (0.02/0.05)	(32), 0.03 (0.02/0.05)	(49), 0.03 (0.01/0.06)	0.277
Creatine kinase, U/l						
- Before PCI (n), Median (IQR)	(1169), 79 (52/115)	(276), 84 (56/119)	(280), 87 (58/117)	(296), 83 (52/117)	(317), 68 (46/105)	0.560
- Day 1 after PCI (n), Median (IQR)	(1161), 66 (46/98)	(276), 69 (48/101)	(280), 68 (49/107)	(296), 69 (45/97)	(317), 62 (43/91)	0.878
Re-catheterizations						0.291
- < 30 days, n (%)	610 (7)	130 (6)	125 (7)	166 (8)	189 (8)	
- 30days– 6 months, n (%)	387 (5)	102 (5)	84 (4)	111 (5)	90 (4)	
- 6 months– 1 year, n (%)	258 (3)	79 (4)	62 (3)	59 (3)	58 (3)	
- > 1 year, n (%)	504 (6)	136 (7)	142 (8)	134 (6)	92 (4)	

Significant differences according to quartiles:

^‡^ = Q1 vs. Q4,

^¶^ = Q2 vs. Q4,

^#^ = Q3 vs. Q4.

Quartile 1 = <58 years;

Quartile 2 = 58–65 years;

Quartile 3 = 66–73 years;

Quartile 4 = >73 years.

**Abbreviations**:

p = p-value;

VD = vessel disease.

### OCT substudy

**Fig 2:**
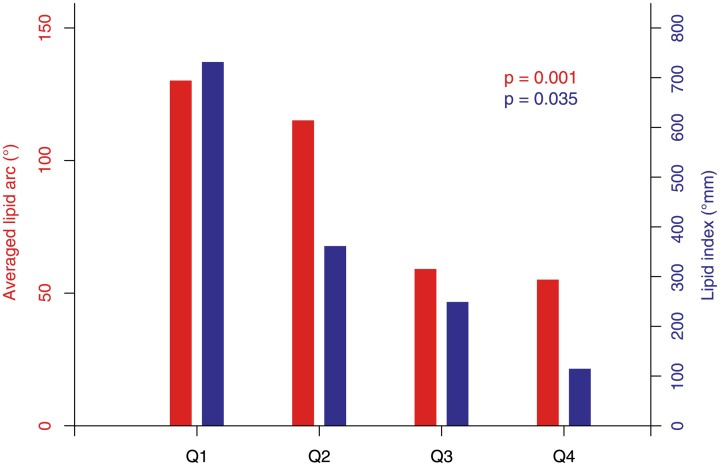
Averaged lipid arc and lipid index. Averaged lipid arc and lipid index stratified according to age into four quartiles.

Lipid plaques are most frequent in the youngest patient group. Incidence and size of lipid plaques are decreasing with age (Fig 2). In contrast, the presence of calcification as well as the total amount of plaque burden is increasing with age. Accordingly, the highest amount of calcified plaques, as well as of total plaque burden, was detected in the oldest patient group (Table 4).

**Table 4:** OCT analysis.

	All, n = 40	Quartile 1, n = 10	Quartile 2, n = 10	Quartile 3, n = 10	Quartile 4, n = 10	p
Type of plaque						
- LP/ FP/CP (n)	17/12/11	9/1/0	6/3/1	0/6/4	2/2/6	<0.0001 †‡§
Lipid content						
- Present (n)	27	10	6	5	6	0,081
- Averaged lipid arc (°, Mean±SD)	97±45	130±36	115±38	59±18	55±14	<0.0001 †‡§¶
- Lipid index (°mm, Mean±SD)	514±522	729±753	360±392	248±279	114±167	0.017
Calcification						
- Present (n)	16	2	2	4	8	0,019 ‡¶
- Averaged calcification arc (°, Mean±SD)	53±15	53±13	44±14	62±21	51±12	0.566
- Calcification index (°mm, Mean±SD)	55±15	54±13	47±14	64±22	53±13	0.599
Total plaque extent						
- Averaged arc of total plaque (°, Mean±SD)	174±52	156±53	146±43	193±32	197±62	0.054
- Total plaque index, (°mm, Mean±SD)	2314±1894	1989±1458	1599±1142	2222±1238	3387±2797	0.155
- Maximal area (mm^2^, Mean±SD)	3.79±1.53	3.45±2.01	2.68±0.88	4.11±0.96	4.87±1.35	0.006 ¶
- Maximal thickness (mm2, Mean±SD)	0.79±0.21	0.68±0,17	0.64±0.13	0.87±0.18	0.94±0.19	0.001 †‡§¶

Significant differences according to quartiles:

^†^ = Q1 vs. Q3,

^‡^ = Q1 vs. Q4,

^§^ = Q2 vs. Q3,

^¶^ = Q2 vs. Q4.

Quartile 1 = <58 years;

Quartile 2 = 58–65 years;

Quartile 3 = 66–73 years;

Quartile 4 = >73 years.

**Abbreviations**:

CP = calcified plaque;

FP = fibrous plaque;

LP = lipid plaque;

p = p-value.

### Mortality

During the observation period 2,009 (24%) patients died. The most common causes of death were cardiovascular (56%) and cancer (18%). Of course, mortality rate rose with increasing age at a death rate from 13% (in patients aged less than 58 years) up to 44% (in elder patients aged over 73 years). Interestingly, the incidence of cardiovascular death and death from cancer was similar in all groups of age (Table 5).

**Table 5:** Cause of death **.

	All, n = 8,342	Quartile 1, n = 2,046	Quartile 2, n = 1,899	Quartile 3, n = 2,090	Quartile 4, n = 2,307	p
Total death, n (%)	2,009 (24)	257 (13)	351 (19)	516 (25)	885 (38)	<0,0001 *†‡§¶#
Cardiovascular, n (%)	1,132 (56)	132 (52)	200 (57)	276 (53)	524 (59)	0,063
Cancer, n (%)	365 (18)	42 (16)	68 (19)	106 (21)	149 (17)	0,269
Gastrointestinal, n (%)	45 (2)	8 (13)	7 (2)	12 (2)	18 (2)	0,758
Respiratory, n (%)	86 (4)	11 (4)	12 (3)	25 (5)	38 (4)	0,792
Non-natural, n (%)	73 (4)	14 (6)	9 (3)	16 (3)	34 (4)	0,252
Other, n (%)	308 (16)	50 (19)	55 (16)	81 (16)	122 (14)	0,166

** Percentages of total death refer to the number of all patients in the corresponding study group. Percentages of different causes of death refer to the number of total deaths in the corresponding study group.

Significant differences according to quartiles:

* = Q1 vs. Q2,

^†^ = Q1 vs. Q3,

^‡^ = Q1 vs. Q4,

^§^ = Q2 vs. Q3,

^¶^ = Q2 vs. Q4,

^#^ = Q3 vs. Q4.

Quartile 1 = <58 years;

Quartile 2 = 58–65 years;

Quartile 3 = 66–73 years;

Quartile 4 = >73 years.

**Abbreviations**:

p = p-value.

### Univariate and multivariate results

Table 6 lists the results of the univariate analysis. In a multivariate model (Table 7) age was the most important predictor of survival. The corresponding Hazard ratio (HR) of 1.043 means that an additional year of age increases the Hazard by 4.3%. Furthermore, the presence of peripheral vascular disease, IDDM, and NIDDM, prior MI, central vascular disease, and prior CABG increase the Hazard as well. In contrast, treated hyperlipidemia, family history, and prior PCI reduce the Hazard. Interestingly, a positive family history is associated with a better survival. This phenomenon may result from the fact that especially younger patients are characterized by a positive family history, and younger patients per se have a better survival than elder patients.

**Table 6:** Univariate results.

	HR	low95	up95	p
Age	1.039	1.034	1.045	<0.0001
Gender	1.068	0.956	1.193	0.242
Height	0.553	0.303	1.012	0.055
Weight	0.995	0.992	0.999	0.015
Body mass index	0.983	0.969	0.997	0.017
Heart rate, bpm	1.015	1.011	1.018	<0.0001
Arterial hypertension	1.041	0.930	1.165	0.482
Systolic blood pressure, mmHg	0.998	0.995	1.001	0.285
Diastolic blood pressure, mmHg	0.988	0.983	0.993	<0.0001
Diabetes mellitus—NIDDM	1.277	1.124	1.452	<0.0001
Diabetes mellitus—IDDM	1.564	1.321	1.853	<0.0001
Treated hyperlipidemia	0.799	0.716	0.891	<0.0001
Smoker—current	0.868	0.749	1.006	0.059
Smoker—previous	1.116	0.988	1.262	0.078
Family history	0.702	0.596	0.826	<0.0001
Central vascular disease	1.494	1.303	1.713	<0.0001
Peripheral vascular disease, n (%)*	2.009	1.751	2.305	<0.0001
Prior myocardial infarction, n (%)*	1.219	1.088	1.365	0.001
Prior PCI, n (%)*	1.009	0.883	1.153	0.892
Prior coronary artery bypass graft, n (%)*	1.277	1.089	1.498	0.003
PM, n (%)*	1.620	1.295	2.027	<0.0001
CRT, n (%)	2.452	1.167	5.154	0.018
NYHA class	1.236	1.114	1.371	<0.0001
CCS class	0.894	0.835	0.956	0.001
ACE inhibitor	1.078	0.966	1.202	0.179
ARB	1.048	0.914	1.202	0.504
ACE inhibitor or ARB	1.138	1.014	1.276	0.028
Aldosteron-antagonist	2.129	1.815	2.497	<0.0001
Beta-blocker	0.938	0.838	1.050	0.265
Statin	0.826	0.736	0.927	0.001
Aspirin	0.968	0.722	1.298	0.828
P2Y12 inhibitor	3.019	1.436	6.349	0.004
Dual antiplatelet therapy	1.138	0.880	1.473	0.324
Calcium-channel blocker	1.071	0.947	1.211	0.272
Nitrates	1.188	1.050	1.344	0.006
Vessel disease	1.026	0.965	1.092	0.409
Chronic total occlusion	1.177	1.033	1.342	0.014
Number of procedures	0.938	0.873	1.007	0.079
Re-catheterizations	0.945	0.912	0.980	0.002

**Abbreviations**:

ACE = angiotensin-converting-enzyme;

ARB = angiotensin receptor blocker;

CCS = canadian cardiovascular society;

CRT = cardiac resynchronization therapy;

HR = hazard ratio;

IDDM = insulin dependent diabetes mellitus;

Low95 = lower bound for 95% confidence interval;

LVEF = left ventricular ejection fraction;

NIDDM = non insulin dependent diabetes mellitus;

NT-proBNP = n-terminal pro-brain natriuretic peptide;

NYHA = new york heart association;

p = p-value;

PCI = percutaneous coronary interventio;

PM = pacemaker;

Up95 = upper bound for 95% confidence interval.

**Table 7:** Multivariate results.

	HR	low95	up95	p
Age	1.033	1.024	1.041	<0.0001
Peripheral vascular disease	1.886	1.547	2.300	<0.0001
Heart rate	1.011	1.007	1.016	<0.0001
Aldosterone antagonists	1.405	1.121	1.761	0.003
Treated hyperlipidemia	0.805	0.679	0.953	0.012
NYHA class	1.282	1.096	1.500	0.002
Diastolic blood pressure	0.992	0.985	0.998	0.013
CCS class	0.881	0.795	0.978	0.170
Pacemaker	1.382	1.030	1.855	0.031
Re-catheterization	0.944	0.895	0.996	0.034
Family history	0.795	0.634	0.996	0.046

**Abbreviations**:

CCS = canadian cardiovascular society;

HR = hazard ratio;

Low95 = lower bound for 95% confidence interval;

NYHA = new york heart association;

p = p-value;

Up95 = upper bound for 95% confidence interval.

### Relative survival

**Fig 3:**
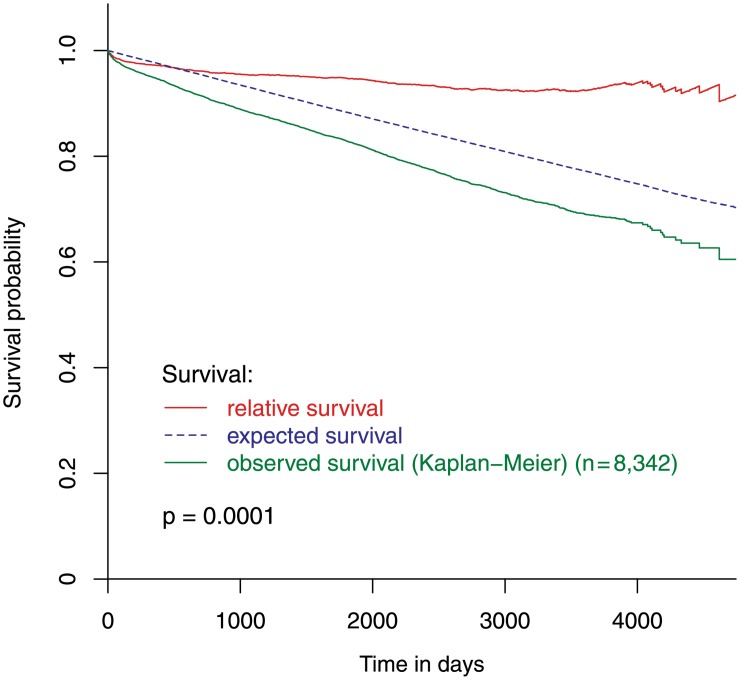
Observed, expected and relative survival of all study-patients. The observed survival of all study-patients, the expected survival of the sex- and age-matched general population, and the resulting relative survival of all study-patients.

The observed patient survival was worse than the expected survival based on age- and gender-matched background population (p<0.001). The difference between these two groups continuously increased until the end of the observed time period. Hence, the relative survival curve starts almost horizontally and only slightly decreases afterwards (Fig 3).

### Baseline and angiographic characteristics according to age

Patients were stratified according to their age by using quartiles. Quartile 1 (Q1: <58 years) comprised 2,046 patients, Quartile 2 (Q2: 58–65 years) 1,899 patients, Quartile 3 (Q3: 66–73 years) 2,090 patients, and Quartile 4 (Q4: >73 years) 2,307 patients. Baseline characteristics are given in Table 1. Interestingly, Quartile 1 includes the highest proportion of patients with positive family history (23%). Younger patients are highly characterized by having more current smokers and having a higher level of total and LDL cholesterol, whereas elder patients have a higher incidence of diabetes, elevated blood pressure, and chronic renal impairment (Table 1). Table 3 shows the angiographic characteristics according to age groups. Of note, the severity of CAD was distributed relatively homogenous across age groups with about 50% of patients suffering from a one-vessel disease and about 25% of patients suffering from a two- and three-vessel disease.

### Mortality according to age

In all quartiles cardiovascular death was the most common cause of death. As expected, most people died in Quartile 4, however the proportion between cardiovascular death and other causes of death was similar in each group. The exact figures are shown in Table 5.

### Observed and relative survival according to age

**Fig 4:**
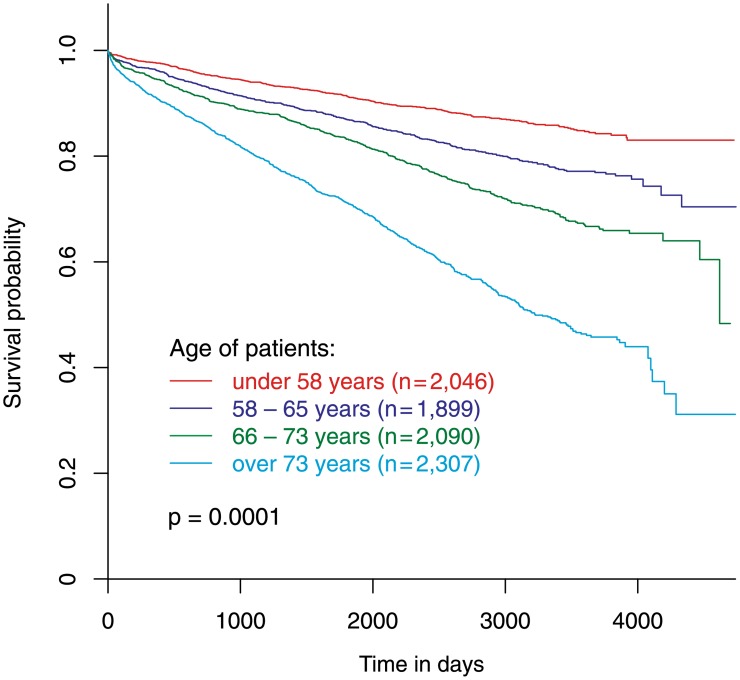
Observed survival according to age quartiles. Observed survival of patients stratified according to age into four quartiles.

In a multivariate Cox regression model age was the most important predictor of survival (Table 7). Large differences in observed survival can be found between different age groups (p = 0.001) (Fig 4). Even the second age quartile has a statistically significant worse survival than the first age quartile (the youngest patient group) with a hazard ratio of 1.59 (p<0.001). Third age quartile and forth age quartile show hazard ratios of 2.29 (p<0.001) and 4.29 (p<0.001), respectively.

**Fig 5:**
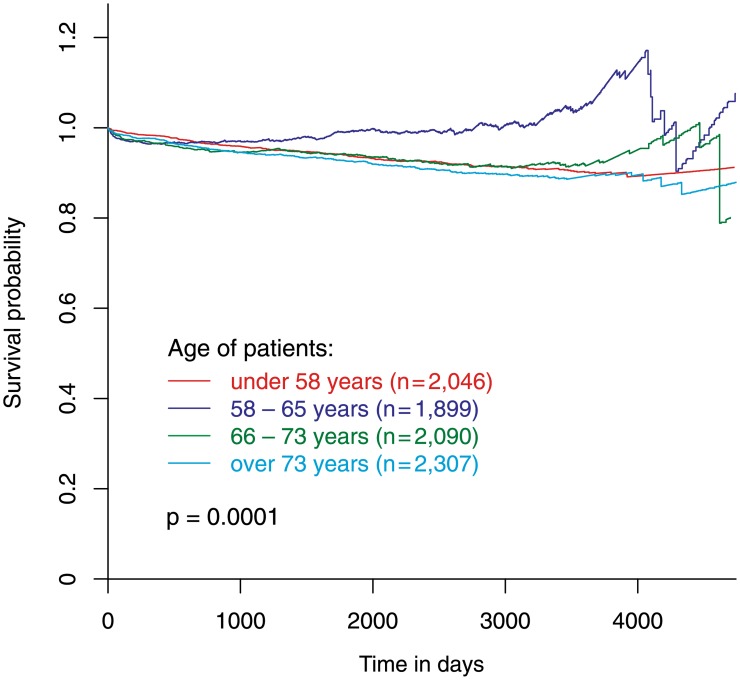
Relative survival according to age quartiles. Relative survival of patients stratified according to age into four quartiles.

The relative survival of the younger population (Q1: <58 years, 2,046 patients), the elder population (Q3: 66–73 years, 2,090 patients) and the very old population (Q4: >73 years, 2,307 patients) was similar. The relative survival of mid-aged patients (Q2: 58–65 years, 1,899 patients) was significantly better (p = 0.001) than the relative survival of all other patient groups (Fig 5).

## Discussion

Our analysis indicates that patients with stable CAD, who undergo elective PCI, have a slightly worse survival (observed) than the general population (expected survival). In our patients mortality-risk continuously increased with age similar to the general population. When stratifying patients according to their age, the relative survival of mid-aged patients between 58 and 65 years was better than the relative survival of younger and elder patient groups (Fig 5). The relative survival of elder patients—between 66 and 73 years and above 73 years—was similar to the relative survival of younger patients below 58 years suggesting a similar benefit from PCI in these patient groups (Fig 5). These differences are paralleled by different profiles of cardiovascular risk factors and consecutive different patterns of plaque morphology.

### Cardiovascular risk factors and plaque morphology

Cardiovascular risk factors differ considerably between various groups of age. Younger patients are highly characterized by having more current smokers and having a higher level of total and LDL cholesterol. This risk profile in younger patients is also documented by other authors [22,23]. Their higher genetic predisposition for coronary artery disease is reflected by a higher incidence of positive family history. In contrast, elder patients are characterized by a distinct pattern of cardiovascular risk factors with a higher incidence of diabetes, elevated blood pressure, and chronic renal impairment. However, they have lower total and LDL-cholesterol levels and are less frequently current smokers. A similar distribution of risk factors was recently documented in patients with ACS [24].

Recent analyses showed, that specific risk factors cause certain plaque characteristics. Current smokers are more likely to have lipid plaques and OCT-defined vulnerable plaques [25]. Similarly, LDL cholesterol levels are positively correlated to the extent of lipid plaques [26]. On the other hand, chronic renal impairment and diabetes are accompanied by an increased plaque burden with higher calcium content [27,28]. These findings confirm the results of our OCT sub-analysis: younger patients, who were more frequently smokers and had elevated total and LDL-cholesterol levels, had higher rates of large lipid plaques compared to elder patients. These plaques, also called fibroatheroma, include thin-cap atheroma and may cause acute coronary syndrome when rupturing. Elder patients with higher rates of diabetes and chronic renal impairment were characterized by a considerably lower rate of large lipid plaques but a higher rate of calcified plaques and an increased plaque burden.

### Plaque morphology and age

Age-dependent differences in plaque composition have already been described by some authors: several analyses indicate that plaque burden and calcium content increase with age [24,29,30]. These findings are confirmed in our analysis. Moreover, the incidence of thin-cap fibroatheroma (TCFA), defined as a necrotic core (lipid plaque) situated next to the vessel lumen, may change with age. In the PROSPECT study, which used virtual-histology intravascular ultrasound (VH-IVUS) for identification of a necrotic core (lipid pool), thickcap-FA increased in patients above 65 years, whereas TCFA were, in trend, more frequent in patients below 65 years [24]. However, VH-IVUS has been reported to overestimate the extent of a lipid plaque [31,32]. A fact, that may especially apply for plaques with multiple components, typically found in the elderly. In contrast, OCT has a higher sensitivity (90% to 92%) to identify lipid-rich plaques [33]. Accordingly, OCT analyses found out that thick-cap FA consists of considerably smaller lipid pools but a higher amount of fibrosis and calcification area compared to TCFA [34]. OCT criteria for a TCFA include the presence of a lipid plaque with an arc > 90° and a thin fibrous cap [35]. Recent data [36] documented that an arc of lipid >80° can excellently identify TCFA (area under the curve 0.86). In accordance with these findings, we found significant lipid plaques—suspicious to be a TCFA—mainly in younger (<58 years) and mid-aged patients (58–65 years). Elder patients predominantly had smaller lipid plaques, not fulfilling the criteria for a TCFA, embedded in large mixed plaques with fibrosis and frequent calcific components. Two reports, that compared plaque morphology in younger and elder patient groups, found similar results [36,37].

### Possible explanations for age-dependent differences in relative survival

In younger patients a more aggressive course of disease can be assumed, as their coronary artery disease occurs earlier during life-time. Due to a high rate of positive family history a genetic disposition is likely in many of these patients [38,39]. Compared to elder patients, higher cholesterol levels and a higher incidence of smoking result in larger lipid plaques suspicious to be TCFA. Few data reported that this patient group has a worse long-term survival [23,40], as also confirmed by our analysis.

In elder patients the greater plaque burden indicates a more advanced stage of disease and is accompanied with a higher incidence of comorbidities like peripheral vascular disease, central vascular disease, prior MI, and prior CABG. This finding is well documented in other large observational studies [41,42], presumably resulting from a long history of various cardiovascular risk factors, and may enlarge the survival gap between CAD patients and the general population. Despite these unfavorable factors on long-term outcome, elder patients have a similar relative survival compared to our younger patient group. Accordingly, elective PCI can be suggested to be a useful tool to treat these patients. Another factor may contribute to the better relative survival in these patients. The acute coronary syndrome (ACS) represents a major complication during the career of a patient with CAD. Current guidelines support the use of an early invasive strategy (early in-hospital coronary angiography followed by revascularization, if appropriate) for patients with moderate-risk and high-risk NSTEMI, rather than a selective approach based on recurrent symptoms or evidence of ongoing ischemia [43,44]. Interestingly, the risk for adverse events with an early instead of a selective invasive approach decreases with age [45,46]. Accordingly, patients aged above 75 years gain the greatest survival benefit from the use of an early invasive approach as demonstrated recently in an analysis of a large database including more than 11,000 patients [47]. However, the same analysis showed that an age above 75 years was the strongest negative predictor for the use of cardiac catheterization. The underestimation of risk by physicians was the most common reason for choosing a conservative strategy. During the long-term follow-up of our patients this factor may worsen the outcome in elder patient groups compared to the younger.

Mid-aged patients between 58 and 65 years have a less aggressive severity of disease compared to younger patients, a less advanced stage of disease and is accompanied with a higher incidence of comorbidities. These patients may gain the most benefit from treatment of their lipid plaques. Compared with medical treatment alone, PCI reduces the subsequent risk of spontaneous MI in patients with CAD [48,49]. As the incidence of STEMI has an age-peek between 55 and 70 years and rapidly decreases thereafter [50], patients in this group of age, with the highest risk to suffer from a STEMI, may benefit most from elective PCI. Our data support this suggestion.

### Cause of death

The cardiovascular mortality of 56% in our study is in accordance with other studies, which report death-rates due to cardiac causes (not cardiovascular) of below or equal to 50% in patients with stable coronary artery disease [51,52]. When comparing the main causes of death including cardiovascular causes, cancer, gastro-intestinal diseases, diseases of the respiratory system, non-natural and other causes between our study population and the general population [21], the distribution of these causes is rather similar except the following two: Interestingly, less study patients died from cancer compared to the general population (18% versus 26%). One explanation for this finding may be the fact that, not surprisingly, our study population more frequently died from cardiovascular reasons, and therefore had a lower chance to experience cancer compared to the general population. In addition, the statin therapy in patients with CAD may contribute to the lower rate of cancer death [53]. Similar results, a lower death rate from cancer and a higher rate of cardiovascular deaths were reported by the authors of the GENetic DEterminants of Restenosis (GENDER) study, who also compared CAD patients after elective PCI and the general population [54].

### Limitations

As a limitation of our analysis our control group, the general population of Austria, also includes patients with stable CAD. Some of these patients do not seek help from a doctor and others are treated medically and are not sent to the catheter laboratory. These patients may have a worse prognosis to patients treated in the catheter laboratory and compared to the general population. Moreover, some subjects in the general population also underwent PCI in other catheter laboratories. This part of the control group can also be suggested to have a worse prognosis than the general population and a similar prognosis to our study population. Despite of these patients, the mortality rate of our patient population was slightly higher compared to the general population. However, the main message from our analysis results from the comparison of the relative survival between different groups of age. This comparison is not influenced by the background noise of CAD patients in the general population as this background noise can be suggested to be similar in all groups. In addition, the long follow-up includes patients with constant changes in the therapeutic and interventional strategy. On average, medical therapy of our patients is comparable to treatment regimen in studies dedicated to provide optimized medical treatment to these patients [51]. Eighty percent of our patients had been treated with drug-eluting stents and only 20% received bare-metal stents. It can not be excluded that a higher rate of drug-eluting-stents may slightly reduce the survival disadvantage of patients who undergo elective PCI compared to the total population. Accordingly, a potential improvement in survival may also slightly reduce the relative survival benefit of mid-aged patients compared to younger and elder patients. Upcoming new treatment options for additional lipid-lowering may have a more important impact. Our analysis emphasizes the necessity for therapeutic improvement.

### Clinical implications

Younger patients below 58 years (median 52 years) with stable coronary artery disease suffer from a more aggressive type of disease with higher total and LDL cholesterol levels and larger lipid plaques than elder patients. More aggressive lipid lowering strategies should be implemented especially in these patients to improve survival. Further studies will evaluate if screening for lipid plaques (non-invasive or invasive) will be reasonable in these patients. Elder patients suffer from a distinct type of coronary artery disease with higher blood pressure levels, higher rates of diabetes and chronic renal impairment. These patients are characterized by fewer and smaller lipid plaques but more calcification and greater plaque burden associated with late complications like peripheral vascular disease, central vascular disease, or prior MI. Although a large network meta-analysis including more than 90,000 mid-aged patients (median age 62, interquartile range 51/69) provided evidence for improved survival with new generation drug eluting stents compared with medical treatment alone [55], the benefit of PCI in elder patients is unclear. Our analysis clearly demonstrate that elder patients with stable CAD who undergo PCI have a similar relative survival like younger patients. Mid-aged CAD patients have a similar survival compared to the age-matched general population and a slightly better outcome than younger and elder patient groups. These patients can be suggested to benefit most from elective PCI, as they are at high risk to experience a STEMI due to a higher probability to have a vulnerable thin-cap atheroma and elective PCI may be an effective treatment to prevent this event [48,49].
